# Supplementary material for: Global distribution of sporadic sapovirus infections: A systematic review and meta-analysis
Source: PLoS One. 2021 Aug 19;16(8):e0255436. doi: 10.1371/journal.pone.0255436 (PMC8376006; doi:10.1371/journal.pone.0255436)
Supplement: S1 Appendix — (DOCX) [file pone.0255436.s001.docx]

| First author | Article title | Journal | Year | Country | Development Index | Setting(s) | Age(s) | Method of Detection | # of AGE cases tested for sapovirus | # of sapovirus positive AGE cases | # of non-AGE controls tested for sapovirus | # of sapovirus positive non-AGE controls | # of cases sequenced | % GI among sequenced cases | % GII among sequenced cases | % GIV among sequenced cases | % GV among sequenced cases | # of controls sequenced | % GI among sequenced controls | % GII among sequenced controls | % GIV among sequenced controls | % GV among sequenced controls |
| --- | --- | --- | --- | --- | --- | --- | --- | --- | --- | --- | --- | --- | --- | --- | --- | --- | --- | --- | --- | --- | --- | --- |
| Acacio | Risk Factors for Death Among Children 0-59 Months of Age with Moderate-To-Severe Diarrhea in Manhiça District, Southern Mozambique | BMC Infectious Diseases | 2019 | Mozambique | HMD | Mixed | <5 years | Conventional PCR | 821 | 12 |  |  |  |  |  |  |  |  |  |  |  |  |
| Amar | Detection by PCR Of Eight Groups of Enteric Pathogens In 4,627 Faecal Samples: Re-Examination of The English Case-Control Infectious Intestinal Disease Study (1993-1996) | European Journal of Clinical Microbiology and Infectious Diseases | 2007 | United Kingdom | Developed | Community | <5 years, ≥5 years | Conventional PCR | 2422 | 92 | 2205 | 42 |  |  |  |  |  |  |  |  |  |  |
| Aragao | Norovirus Diversity in Diarrheic Children from An African-Descendant Settlement in Belém, Northern Brazil | PLoS ONE | 2013 | Brazil | LMD | Community | <5 years, ≥5 years | Conventional PCR | 81 | 2 |  |  | 2 | 50% | 50% | 0% | 0% |  |  |  |  |  |
| Axelrad | The Distribution of Enteric Infections Utilizing Stool Microbial Polymerase Chain Reaction Testing in Clinical Practice | Digestive Diseases and Sciences | 2018 | United States | Developed | Mixed | Mixed | RT-qPCR | 13231 | 226 |  |  |  |  |  |  |  |  |  |  |  |  |
| Beal | A Gastrointestinal PCR Panel Improves Clinical Management and Lowers Health Care Costs | Journal of Clinical Microbiology | 2018 | United States | Developed | Inpatient | Mixed | RT-qPCR | 241 | 9 |  |  |  |  |  |  |  |  |  |  |  |  |
| Becker-Dreps | Etiology of Childhood Diarrhea After Rotavirus Vaccine Introduction: A Prospective, Population-Based Study in Nicaragua | The Pediatric Infectious Disease Journal | 2014 | Nicaragua | LMD | Community | <5 years | RT-qPCR | 337 | 56 | 106 | 2 | 22 | 41% | 59% | 0% | 0% |  |  |  |  |  |
| Biscaro | Detection and Molecular Characterization of Enteric Viruses in Children with Acute Gastroenteritis in Northern Italy | Infection, Genetics and Evolution | 2018 | Italy | Developed | Inpatient | Mixed | Conventional PCR | 510 | 9 |  |  |  |  |  |  |  |  |  |  |  |  |
| Bozkurt | Eight Different Viral Agents in Childhood Acute Gastroenteritis | Turkish Journal of Pediatrics | 2015 | Turkey | LMD | Mixed | Mixed | Conventional PCR | 240 | 5 |  |  |  |  |  |  |  |  |  |  |  |  |
| Bucardo | Susceptibility of Children to Sapovirus Infections, Nicaragua, 2005-2006 | Emerging Infectious Diseases | 2012 | Nicaragua | LMD | Inpatient, Community | <5 years | Conventional PCR | 205 | 25 | 87 | 8 |  |  |  |  |  |  |  |  |  |  |
| Bucardo | Predominance of Norovirus and Sapovirus In Nicaragua After Implementation of Universal Rotavirus Vaccination | PLoS One | 2014 | Nicaragua | LMD | Inpatient, Outpatient | <5 years | RT-qPCR | 330 | 57 |  |  | 44 | 59% | 36% | 5% | 0% |  |  |  |  |  |
| Buesa | Molecular Epidemiology of Caliciviruses Causing Outbreaks and Sporadic Cases of Acute Gastroenteritis in Spain | Journal of clinical microbiology | 2002 | Spain | Developed | Mixed | <5 years | Conventional PCR | 310 | 4 |  |  |  |  |  |  |  |  |  |  |  |  |
| Calderaro | Contribution of The FilmArray((R)) Gastrointestinal Panel in The Laboratory Diagnosis of Gastroenteritis in A Cohort of Children: A Two-Year Prospective Study | International Journal of Medical Microbiology | 2018 | Italy | Developed | Mixed | <5 years, ≥5 years | RT-qPCR | 1716 | 115 |  |  |  |  |  |  |  |  |  |  |  |  |
| Chaimongkol | Molecular Characterization of Norovirus Variants and Genetic Diversity of Noroviruses and Sapoviruses In Thailand | Journal of Medical Virology | 2014 | Thailand | LMD | Inpatient | <5 years | Conventional PCR | 567 | 7 |  |  | 7 | 100% | 0% | 0% | 0% |  |  |  |  |  |
| Chan-It | Emergence of A New Norovirus GII.6 Variant in Japan, 2008-2009 | Journal of Medical Virology | 2012 | Japan | Developed | Outpatient | Mixed | Conventional PCR | 187 | 2 |  |  |  |  |  |  |  |  |  |  |  |  |
| Chanit | Intergenogroup Recombinant Sapovirus In Japan, 2007-2008 | Emerging Infectious Diseases | 2009 | Japan | Developed | Outpatient | Mixed | Conventional PCR | 477 | 19 |  |  | 19 | 21% | 0% | 79% | 0% |  |  |  |  |  |
| Cheng | Epidemiological Study of Human Calicivirus Infection in Children with Gastroenteritis in Lanzhou from 2001 To 2007 | Archives of Virology | 2010 | China | LMD | Inpatient | <5 years | Conventional PCR | 1195 | 10 |  |  | 10 | 50% | 50% | 0% | 0% |  |  |  |  |  |
| Chen | Severe Viral Gastroenteritis in Children After Suboptimal Rotavirus Immunization in Taiwan | The Pediatric Infectious Disease Journal | 2013 | Taiwan | LMD | Inpatient | Mixed | Conventional PCR | 755 | 6 |  |  |  |  |  |  |  |  |  |  |  |  |
| Chen | Viral Agents Associated with Acute Diarrhea Among Outpatient Children in Southeastern China | The Pediatric Infectious Disease Journal | 2013 | China | LMD | Outpatient | <5 years | Conventional PCR | 811 | 23 |  |  |  |  |  |  |  |  |  |  |  |  |
| Chen | Molecular Detection and Phylogenetic Analysis of Human Parechovirus In Individuals with Acute Diarrhea and Healthy Controls in Guangzhou, China | Journal of Medical Virology | 2018 | China | LMD | Outpatient | Mixed | Conventional PCR | 430 | 5 | 28 | 0 |  |  |  |  |  |  |  |  |  |  |
| Chen | Prevalence of Enteropathogens In Outpatients with Acute Diarrhea from Urban and Rural Areas, Southeast China, 2010-2014 | The American Journal of Tropical Medicine and Hygiene | 2019 | China | LMD | Outpatient | Mixed | Conventional PCR | 3060 | 47 |  |  |  |  |  |  |  |  |  |  |  |  |
| Chhabra | Etiology of Viral Gastroenteritis in Children <5 Years of Age in The United States, 2008-2009 | Journal of Infectious Diseases | 2013 | United States | Developed | Mixed | <5 years | RT-qPCR | 782 | 42 | 499 | 21 |  |  |  |  |  |  |  |  |  |  |
| Cunliffe | Healthcare-Associated Viral Gastroenteritis Among Children in A Large Pediatric Hospital, United Kingdom | Emerging Infectious Diseases | 2010 | United Kingdom | Developed | Inpatient | Mixed | Conventional PCR | 576 | 27 |  |  |  |  |  |  |  |  |  |  |  |  |
| Dey | Prevalence of Sapovirus Infection Among Infants and Children with Acute Gastroenteritis in Dhaka City, Bangladesh During 2004-2005 | Journal of Medical Virology | 2007 | Bangladesh | HMD | Inpatient | <5 years | Conventional PCR | 917 | 25 |  |  | 25 | 100% | 0% | 0% | 0% |  |  |  |  |  |
| Dey | Seasonal Pattern and Genotype Distribution of Sapovirus Infection in Japan, 2003-2009 | Epidemiology and infection | 2012 | Japan | Developed | Mixed | Mixed | Conventional PCR | 3232 | 123 |  |  | 123 | 85% | 3% | 12% | 0% |  |  |  |  |  |
| Diez-Valcarce | Prevalence and Genetic Diversity of Viral Gastroenteritis Viruses in Children Younger Than 5 Years of Age in Guatemala, 2014-2015 | Journal of Clinical Virology | 2019 | Guatemala | HMD | Inpatient, Outpatient | <5 years | RT-qPCR | 471 | 33 |  |  | 13 | 15% | 54% | 31% | 0% |  |  |  |  |  |
| Doll | Temporal Changes in Pediatric Gastroenteritis After Rotavirus Vaccination in Quebec | The Pediatric Infectious Disease Journal | 2016 | Canada | Developed | Inpatient | <5 years | RT-qPCR | 705 | 51 |  |  |  |  |  |  |  |  |  |  |  |  |
| Fioretti | Occurrence of Human Sapoviruses In Wastewater and Stool Samples in Rio De Janeiro, Brazil | Journal of Applied Microbiology | 2016 | Brazil | LMD | Inpatient, Outpatient | Mixed | RT-qPCR | 341 | 12 |  |  | 7 | 71% | 14% | 0% | 14% |  |  |  |  |  |
| Gao | Human Calicivirus Occurrence Among Outpatients with Diarrhea in Beijing, China, Between April 2011 And March 2013 | Journal of Medical Virology | 2015 | China | LMD | Outpatient | Mixed | Conventional PCR | 3832 | 24 |  |  |  |  |  |  |  |  |  |  |  |  |
| Gonzalez-Galan | High Prevalence of Community-Acquired Norovirus Gastroenteritis Among Hospitalized Children: A Prospective Study | Clinical Microbiology and Infection | 2011 | Spain | Developed | Inpatient | <5 years | Conventional PCR | 399 | 0 |  |  |  |  |  |  |  |  |  |  |  |  |
| Gonzalez | Molecular Epidemiology of Enteric Viruses in Children with Sporadic Gastroenteritis in Valencia, Venezuela | Journal of Medical Virology | 2011 | Venezuela | LMD | Inpatient | <5 years | Conventional PCR | 480 | 12 |  |  | 8 | 75% | 0% | 25% | 0% |  |  |  |  |  |
| Grant | Norovirus and Sapovirus Epidemiology and Strain Characteristics Among Navajo And Apache Infants | PLoS ONE | 2017 | United States | Developed | Community | <5 years | RT-qPCR | 241 | 8 | 343 | 9 | 6 | 67% | 33% | 0% | 0% | 3 | 33% | 33% | 33% | 0% |
| Grytdal | Incidence of Norovirus and Other Viral Pathogens That Cause Acute Gastroenteritis (AGE) Among Kaiser Permanente Member Populations in The United States, 2012-2013 | PLoS ONE | 2016 | United States | Developed | Outpatient | <5 years, ≥5 years | RT-qPCR | 1099 | 19 |  |  |  |  |  |  |  |  |  |  |  |  |
| Gupta | Aetiology Of Childhood Viral Gastroenteritis in Lucknow, North India | The Indian Journal of Medical Research | 2015 | India | HMD | Inpatient, Community | <5 years | Conventional PCR | 278 | 8 |  |  |  |  |  |  |  |  |  |  |  |  |
| Hall | Incidence of Acute Gastroenteritis and Role of Norovirus, Georgia, USA, 2004-2005 | Emerging Infectious Diseases | 2011 | United States | Developed | Outpatient | <5 years, ≥5 years | Conventional PCR | 572 | 7 |  |  |  |  |  |  |  |  |  |  |  |  |
| Ham | Prevalence of Human Astrovirus In Patients with Acute Gastroenteritis | Annals of Laboratory Medicine | 2014 | South Korea | Developed | Inpatient | Mixed | Conventional PCR | 9597 | 14 |  |  |  |  |  |  |  |  |  |  |  |  |
| Hansman | Genetic Diversity of Norovirus and Sapovirus In Hospitalized Infants with Sporadic Cases of Acute Gastroenteritis in Chiang Mai, Thailand | Journal of Clinical Microbiology | 2004 | Thailand | LMD | Inpatient | <5 years | Conventional PCR | 105 | 5 |  |  | 5 | 60% | 40% | 0% | 0% |  |  |  |  |  |
| Harada | Surveillance of Pathogens in Outpatients with Gastroenteritis and Characterization of Sapovirus Strains Between 2002 And 2007 In Kumamoto Prefecture, Japan | Journal of Medical Virology | 2009 | Japan | Developed | Outpatient | <5 years, ≥5 years | Conventional PCR | 639 | 81 |  |  | 81 | 21% | 12% | 63% | 4% |  |  |  |  |  |
| Harada | A Confirmation of Sapovirus Re-Infection Gastroenteritis Cases with Different Genogroups And Genetic Shifts in The Evolving Sapovirus Genotypes, 2002-2011 | Archives of Virology | 2012 | Japan | Developed | Outpatient | Mixed | Conventional PCR | 728 | 58 |  |  | 58 | 31% | 64% | 0% | 5% |  |  |  |  |  |
| Hassan | Viral Etiology of Acute Gastroenteritis In <2-Year-Old US Children in The Post-Rotavirus Vaccine Era | Journal of the Pediatric Infectious Diseases Society | 2019 | United States | Developed | Mixed | <5 years | RT-qPCR | 330 | 22 | 272 | 9 | 13 | 69% | 31% | 0% | 0% | 7 | 71% | 29% | 0% | 0% |
| Heusinkveld | Potential Causative Agents of Acute Gastroenteritis in Households with Preschool Children: Prevalence, Risk Factors, Clinical Relevance and Household Transmission | European Journal of Clinical Microbiology and Infectious Diseases | 2016 | Netherlands | Developed | Community | <5 years, ≥5 years | RT-qPCR | 357 | 14 | 1486 | 40 |  |  |  |  |  |  |  |  |  |  |
| Iturriza-Gomara | Structured Surveillance of Infectious Intestinal Disease in Pre-School Children in The Community: "The Nappy Study" | Epidemiology and Infection | 2009 | United Kingdom | Developed | Outpatient | <5 years | Conventional PCR | 583 | 74 |  |  |  |  |  |  |  |  |  |  |  |  |
| Japhet | Viral Gastroenteritis Among Children Of 0-5 Years in Nigeria: Characterization of The First Nigerian Aichivirus, Recombinant Noroviruses and Detection of a Zoonotic Astrovirus | Journal of Clinical Virology | 2019 | Nigeria | HMD | Mixed | <5 years | Conventional PCR | 103 | 0 |  |  |  |  |  |  |  |  |  |  |  |  |
| Jin | Viral Agents Associated with Acute Gastroenteritis in Children Hospitalized with Diarrhea in Lanzhou, China | Journal of Clinical Virology | 2009 | China | LMD | Inpatient | <5 years | Conventional PCR | 544 | 6 |  |  | 6 | 83% | 17% | 0% | 0% |  |  |  |  |  |
| Johnsen | Genetic Diversity of Sapovirus Infections in Danish Children 2005-2007 | Journal of Clinical Virology | 2009 | Denmark | Developed | Mixed | <5 years | RT-qPCR | 1104 | 97 |  |  | 91 | 55% | 37% | 0% | 8% |  |  |  |  |  |
| Junquera | Prevalence and Clinical Characteristics of Norovirus Gastroenteritis Among Hospitalized Children in Spain | The Pediatric Infectious Disease Journal | 2009 | Spain | Developed | Inpatient | <5 years | Conventional PCR | 352 | 0 |  |  |  |  |  |  |  |  |  |  |  |  |
| Kambhampati | Active Surveillance for Norovirus in A US Veterans Affairs Patient Population, Houston, Texas, 2015-2016 | Open Forum Infectious Diseases | 2019 | United States | Developed | Inpatient | ≥5 years | RT-qPCR | 147 | 1 | 19 | 0 | 1 | 0% | 0% | 100% | 0% |  |  |  |  |  |
| Khamrin | Genetic Diversity of Noroviruses and Sapoviruses In Children Hospitalized with Acute Gastroenteritis in Chiang Mai, Thailand | Journal of Medical Virology | 2007 | Thailand | LMD | Inpatient | <5 years | Conventional PCR | 248 | 3 |  |  | 3 | 67% | 0% | 33% | 0% |  |  |  |  |  |
| Khamrin | Emergence of New Norovirus Variants and Genetic Heterogeneity of Noroviruses and Sapoviruses In Children Admitted to Hospital with Diarrhea in Thailand | Journal of Medical Virology | 2010 | Thailand | LMD | Inpatient | <5 years | Conventional PCR | 147 | 5 |  |  | 5 | 80% | 20% | 0% | 0% |  |  |  |  |  |
| Khamrin | A Single-Tube Multiplex PCR For Rapid Detection in Feces Of 10 Viruses Causing Diarrhea | Journal of Virological Methods | 2011 | Japan | Developed | Mixed | <5 years | Conventional PCR | 235 | 11 |  |  |  |  |  |  |  |  |  |  |  |  |
| Khamrin | Noroviruses and Sapoviruses Associated with Acute Gastroenteritis in Pediatric Patients in Thailand: Increased Detection of Recombinant Norovirus GII.P16/GII.13 Strains | Archives of Virology | 2017 | Thailand | LMD | Inpatient | <5 years | Conventional PCR | 889 | 6 |  |  | 6 | 33% | 50% | 17% | 0% |  |  |  |  |  |
| Kiseleva | Molecular-Genetic Characterization of Human Rotavirus A Strains Circulating in Moscow, Russia (2009-2014) | Virologica Sinica | 2018 | Russia | LMD | Inpatient | <5 years | RT-qPCR | 429 | 6 | 42 | 2 |  |  |  |  |  |  |  |  |  |  |
| Lasure | Epidemiological Profile and Genetic Diversity of Sapoviruses (SaVs) Identified in Children Suffering from Acute Gastroenteritis in Pune, Maharashtra, Western India, 2007-2011 | Epidemiology and Infection | 2017 | India | HMD | Inpatient | <5 years | Conventional PCR | 778 | 21 | 207 | 4 | 21 | 19% | 52% | 14% | 14% | 4 | 50% | 50% | 0% | 0% |
| Leblanc | The Prevalence of Enteric RNA Viruses in Stools from Diarrheic and Non-Diarrheic People in Southwestern Alberta, Canada | Archives of Virology | 2017 | Canada | Developed | Mixed | Mixed | RT-qPCR | 2281 | 97 | 173 | 2 | 10 | 90% | 10% | 0% | 0% |  |  |  |  |  |
| Lekana-Douki | Molecular Epidemiology of Enteric Viruses and Genotyping of Rotavirus A, Adenovirus and Astrovirus Among Children Under 5 Years Old in Gabon | International Journal of Infectious Diseases | 2015 | Gabon | HMD | Outpatient | <5 years | RT-qPCR | 317 | 30 |  |  |  |  |  |  |  |  |  |  |  |  |
| Lima | Etiology and Severity of Diarrheal Diseases in Infants at The Semiarid Region of Brazil: A Case-Control Study | PLoS Neglected Tropical Diseases | 2019 | Brazil | LMD | Community | <5 years | RT-qPCR | 588 | 23 | 573 | 12 |  |  |  |  |  |  |  |  |  |  |
| Liu | Identification of Norovirus as The Top Enteric Viruses Detected in Adult Cases with Acute Gastroenteritis | The American Journal of Tropical Medicine and Hygiene | 2010 | China | LMD | Inpatient | ≥5 years | Conventional PCR | 503 | 3 |  |  |  |  |  |  |  |  |  |  |  |  |
| Liu | Molecular Detection and Characterization of Sapovirus In Hospitalized Children with Acute Gastroenteritis in The Philippines | Journal of Clinical Virology | 2015 | Philippines | LMD | Inpatient | <5 years | RT-qPCR | 417 | 29 |  |  | 26 | 35% | 58% | 0% | 8% |  |  |  |  |  |
| Liu | Etiological Role and Repeated Infections of Sapovirus Among Children Aged Less Than 2 Years in A Cohort Study in A Peri-Urban Community of Peru | Journal of Clinical Microbiology | 2016 | Peru | LMD | Community | <5 years | RT-qPCR | 299 | 54 | 300 | 17 | 29 | 55% | 31% | 7% | 7% | 16 | 31% | 50% | 0% | 19% |
| Li | Aetiology Of Diarrhoeal Disease and Evaluation of Viral-Bacterial Coinfection in Children Under 5 Years Old in China: A Matched Case-Control Study | Clinical Microbiology and Infection | 2016 | China | LMD | Inpatient | <5 years | RT-qPCR | 461 | 30 | 461 | 19 |  |  |  |  |  |  |  |  |  |  |
| Lorrot | Epidemiology and Clinical Features of Gastroenteritis in Hospitalised Children: Prospective Survey During A 2-Year Period in A Parisian Hospital, France | European Journal of Clinical Microbiology and Infectious Diseases | 2011 | France | Developed | Inpatient | Mixed | Conventional PCR | 457 | 2 |  |  |  |  |  |  |  |  |  |  |  |  |
| Lu | Molecular Epidemiology of Human Calicivirus Infections in Children with Acute Diarrhea in Shanghai: A Retrospective Comparison Between Inpatients and Outpatients Treated B | Archives of Virology | 2014 | China | LMD | Inpatient, Outpatient | <5 years | Conventional PCR | 1110 | 2 |  |  | 2 | 50% | 50% | 0% | 0% |  |  |  |  |  |
| Malasao | Genetic Diversity of Norovirus, Sapovirus, And Astrovirus Isolated from Children Hospitalized with Acute Gastroenteritis in Chiang Mai, Thailand | Journal of Medical Virology | 2008 | Thailand | LMD | Inpatient | <5 years | Conventional PCR | 296 | 10 |  |  | 10 | 80% | 20% | 0% | 0% |  |  |  |  |  |
| Mans | Emerging Norovirus GII.4 2008 Variant Detected in Hospitalised Paediatric Patients in South Africa | Journal of Clinical Virology | 2010 | Thailand | LMD | Inpatient | Mixed | RT-qPCR | 245 | 10 |  |  |  |  |  |  |  |  |  |  |  |  |
| Martinez | Sequence Diversity of Human Caliciviruses Recovered from Children with Diarrhea in Mendoza, Argentina, 1995-1998 | Journal of Medical Virology | 2002 | Argentina | Developed | Mixed | <5 years | Conventional PCR | 149 | 10 |  |  |  |  |  |  |  |  |  |  |  |  |
| Maslin | Detection and Characterization of Human Caliciviruses Associated with Sporadic Acute Diarrhea in Adults in Djibouti (Horn of Africa) | The American Journal of Tropical Medicine and Hygiene | 2008 | Djibouti | HMD | Inpatient | ≥5 years | Conventional PCR | 75 | 3 |  |  |  |  |  |  |  |  |  |  |  |  |
| Monica | Human Caliciviruses In Symptomatic and Asymptomatic Infections in Children in Vellore, South India | Journal of Medical Virology | 2007 | India | HMD | Inpatient, Community | <5 years | Conventional PCR | 850 | 35 | 173 | 6 | 23 | 35% | 65% | 0% | 0% | 6 | 50% | 50% | 0% | 0% |
| Murphy | Evaluation of The BioFire FilmArray® Gastrointestinal Panel in a Midwestern Academic Hospital | European Journal of Clinical Microbiology and Infectious Diseases | 2017 | United States | Developed | Mixed | Mixed | RT-qPCR | 2257 | 45 |  |  |  |  |  |  |  |  |  |  |  |  |
| Nakanishi | Detection of Enteric Viruses in Rectal Swabs from Children with Acute Gastroenteritis Attending the Pediatric Outpatient Clinics in Sapporo, Japan | Journal of Clinical Virology | 2009 | Japan | Developed | Outpatient | Mixed | Conventional PCR | 877 | 15 |  |  |  |  |  |  |  |  |  |  |  |  |
| Nguyen | Diversity of Viruses Associated with Acute Gastroenteritis in Children Hospitalized with Diarrhea in Ho Chi Minh City, Vietnam | Journal of Medical Virology | 2007 | Vietnam | LMD | Inpatient | Mixed | Conventional PCR | 1010 | 8 |  |  | 8 | 88% | 13% | 0% | 0% |  |  |  |  |  |
| Nguyen | Norovirus and Sapovirus Infections Among Children with Acute Gastroenteritis in Ho Chi Minh City During 2005-2006 | Journal of Tropical Pediatrics | 2008 | Vietnam | LMD | Mixed | Mixed | Conventional PCR | 502 | 6 |  |  | 6 | 50% | 50% | 0% | 0% |  |  |  |  |  |
| Nobel | Stool PCR For Gastrointestinal Pathogens in Patients with And Without Immune-Mediated Intestinal Diseases | Digestive Diseases and Sciences | 2018 | United States | Developed | Outpatient | ≥5 years | RT-qPCR | 828 | 11 |  |  |  |  |  |  |  |  |  |  |  |  |
| Okada | Molecular Epidemiology and Phylogenetic Analysis of Sapporo-Like Viruses | Archives of Virology | 2002 | Japan | Developed | Mixed | Mixed | Conventional PCR | 529 | 35 |  |  | 35 | 71% | 26% | 3% | 0% |  |  |  |  |  |
| Olesen | Etiology of Diarrhea in Young Children in Denmark: A Case-Control Study | Journal of Clinical Microbiology | 2005 | Denmark | Developed | Mixed | Mixed | Conventional PCR | 340 | 11 | 619 | 8 |  |  |  |  |  |  |  |  |  |  |
| Osborne | Viral Gastroenteritis in Children in Colorado 2006-2009 | Journal of Medical Virology | 2015 | United States | Developed | Inpatient | Mixed | Conventional PCR | 1105 | 2 |  |  |  |  |  |  |  |  |  |  |  |  |
| Page | Sapovirus Prevalence in Children Less Than Five Years of Age Hospitalised For Diarrhoeal Disease in South Africa, 2009-2013 | Journal of Clinical Virology | 2016 | South Africa | HMD | Inpatient | <5 years | Conventional PCR | 3099 | 238 |  |  |  |  |  |  |  |  |  |  |  |  |
| Pang | Effect of Rotavirus Vaccine on Sapporo Virus Gastroenteritis in Finnish Infants | The Pediatric Infectious Disease Journal | 2001 | Finland | Developed | Community | <5 years | Conventional PCR | 1432 | 132 |  |  |  |  |  |  |  |  |  |  |  |  |
| Phan | Virus Diversity and An Outbreak of Group C Rotavirus Among Infants and Children with Diarrhea in Maizuru City, Japan During 2002-2003 | Journal of Medical Virology | 2004 | Japan | Developed | Mixed | Mixed | Conventional PCR | 236 | 6 |  |  |  |  |  |  |  |  |  |  |  |  |
| Phan | Etiologic Agents of Acute Gastroenteritis Among Japanese Infants and Children: Virus Diversity and Genetic Analysis of Sapovirus | Archives of Virology | 2005 | Japan | Developed | Mixed | Mixed | Conventional PCR | 371 | 17 |  |  | 17 | 76% | 24% | 0% | 0% |  |  |  |  |  |
| Phan | Emergence of Rare Sapovirus Genotype Among Infants and Children with Acute Gastroenteritis in Japan | European Journal of Clinical Microbiology and Infectious Diseases | 2007 | Japan | Developed | Outpatient | Mixed | Conventional PCR | 1154 | 49 |  |  | 49 | 100% | 0% | 0% | 0% |  |  |  |  |  |
| Pitkanen | The Role of The Sapovirus Infection Increased in Gastroenteritis After National Immunisation Was Introduced | Acta Paediatrica | 2019 | Finland | Developed | Inpatient | Mixed | Conventional PCR | 1437 | 48 |  |  | 36 | 44% | 56% | 0% | 0% |  |  |  |  |  |
| Puustinen | Noroviruses as A Major Cause of Acute Gastroenteritis in Children in Finland, 2009-2010 | Scandinavian Journal of Infectious Diseases | 2011 | Finland | Developed | Inpatient, Outpatient | Mixed | Conventional PCR | 195 | 12 |  |  |  |  |  |  |  |  |  |  |  |  |
| Rachakonda | Genetic Diversity of Noroviruses and Sapoviruses In Children with Acute Sporadic Gastroenteritis in New Delhi, India | Journal of Clinical Virology | 2008 | India | HMD | Outpatient | Mixed | Conventional PCR | 226 | 23 |  |  | 23 | 96% | 4% | 0% | 0% |  |  |  |  |  |
| Rovida | Molecular Detection of Gastrointestinal Viral Infections in Hospitalized Patients | Diagnostic Microbiology and Infectious Disease | 2013 | Italy | Developed | Inpatient | Mixed | RT-qPCR | 689 | 8 |  |  |  |  |  |  |  |  |  |  |  |  |
| Saikruang | Detection of Diarrheal Viruses Circulating in Adult Patients in Thailand | Archives of Virology | 2014 | Thailand | LMD | Inpatient | ≥5 years | Conventional PCR | 332 | 0 |  |  |  |  |  |  |  |  |  |  |  |  |
| Sanchez | Epidemiology of Sapovirus Infections in A Birth Cohort in Peru | Clinical Infectious Diseases | 2018 | Peru | LMD | Community | <5 years | RT-qPCR | 877 | 118 | 748 | 73 | 69 | 55% | 26% | 9% | 10% | 36 | 28% | 67% | 6% | 0% |
| Sdiri-Loulizi | Molecular Detection of Genogroup I Sapovirus In Tunisian Children Suffering from Acute Gastroenteritis | Virus Genes | 2011 | Tunisia | LMD | Inpatient, Outpatient | Mixed | Conventional PCR | 788 | 6 |  |  | 6 | 100% | 0% | 0% | 0% |  |  |  |  |  |
| Shen | The 12 Gastrointestinal Pathogens Spectrum of Acute Infectious Diarrhea in A Sentinel Hospital, Shenzhen, China | Frontiers in Microbiology | 2016 | China | LMD | Outpatient | <5 years, ≥5 years | RT-qPCR | 412 | 9 |  |  |  |  |  |  |  |  |  |  |  |  |
| Shioda | Population-Based Incidence Rates of Diarrheal Disease Associated with Norovirus, Sapovirus, And Astrovirus In Kenya | PLoS One | 2016 | Kenya | HMD | Mixed | <5 years, ≥5 years | RT-qPCR | 858 | 44 |  |  |  |  |  |  |  |  |  |  |  |  |
| Silva | Sapovirus In Rectal and Nasopharyngeal Swab Samples of Children with Symptoms of Acute Gastroenteritis | The Pediatric Infectious Disease Journal | 2018 | Brazil | LMD | Inpatient | <5 years, Mixed | RT-qPCR | 102 | 19 |  |  |  |  |  |  |  |  |  |  |  |  |
| Soli | Detection of Enteric Viral and Bacterial Pathogens Associated with Paediatric Diarrhoea In Goroka, Papua New Guinea | International Journal of Infectious Diseases | 2014 | Papua New Guinea | HMD | Inpatient | <5 years | RT-qPCR | 199 | 4 |  |  |  |  |  |  |  |  |  |  |  |  |
| Stockmann | Detection Of 23 Gastrointestinal Pathogens Among Children Who Present with Diarrhea | Journal of the Pediatric Infectious Diseases Society | 2017 | United States | Developed | Mixed | <5 years, ≥5 years | RT-qPCR | 1089 | 64 |  |  |  |  |  |  |  |  |  |  |  |  |
| Supadej | Distribution of Norovirus and Sapovirus Genotypes with Emergence of Nov GII.P16/GII.2 Recombinant Strains in Chiang Mai, Thailand | Journal of Medical Virology | 2018 | Thailand | LMD | Inpatient | Mixed | Conventional PCR | 843 | 18 |  |  | 17 | 88% | 12% | 0% | 0% |  |  |  |  |  |
| Tam | Changes in Causes of Acute Gastroenteritis in The United Kingdom Over 15 Years: Microbiologic Findings From 2 Prospective, Population-Based Studies of Infectious Intestinal Disease | Clinical Infectious Diseases | 2012 | United Kingdom | Developed | Outpatient, Community | Mixed | RT-qPCR | 1656 | 149 |  |  |  |  |  |  |  |  |  |  |  |  |
| Thongprachum | Four-Year Study of Viruses That Cause Diarrhea in Japanese Pediatric Outpatients | Journal of Medical Virology | 2015 | Japan | Developed | Outpatient | <5 years, ≥5 years | Conventional PCR | 2381 | 114 |  |  |  |  |  |  |  |  |  |  |  |  |
| Trang | Detection and Molecular Characterization of Noroviruses and Sapoviruses In Children Admitted to Hospital with Acute Gastroenteritis in Vietnam | Journal of Medical Virology | 2012 | Vietnam | LMD | Inpatient | <5 years | RT-qPCR | 501 | 7 |  |  | 6 | 50% | 50% | 0% | 0% |  |  |  |  |  |
| Varela | Human Sapovirus Among Outpatients with Acute Gastroenteritis in Spain: A One-Year Study | Viruses | 2019 | Spain | Developed | Outpatient | Mixed | RT-qPCR | 2667 | 417 |  |  | 186 | 68% | 30% | 3% | 0% |  |  |  |  |  |
| Wang | Genetic Diversity of Sapovirus In Non-Hospitalized Adults with Sporadic Cases of Acute Gastroenteritis in Shanghai, China | Journal of Clinical Virology | 2014 | China | LMD | Outpatient | ≥5 years | Conventional PCR | 1125 | 42 |  |  | 42 | 83% | 2% | 14% | 0% |  |  |  |  |  |
| Wang | Etiology of Childhood Infectious Diarrhea in A Developed Region of China: Compared to Childhood Diarrhea in A Developing Region and Adult Diarrhea in A Developed Region | PLoS ONE | 2015 | China | LMD | Outpatient | <5 years | Conventional PCR | 1422 | 64 |  |  |  |  |  |  |  |  |  |  |  |  |
| Wang | Burden of Viral Gastroenteritis in Children Living in Rural China: Population-Based Surveillance | International Journal of Infectious Diseases | 2020 | China | LMD | Mixed | <5 years | Conventional PCR | 2306 | 107 |  |  | 80 | 60% | 40% | 0% | 0% |  |  |  |  |  |
| Wu | Surveillance of Pathogens Causing Gastroenteritis and Characterization of Norovirus and Sapovirus Strains in Shenzhen, China, During 2011 | Archives of Virology | 2014 | China | LMD | Outpatient | <5 years, ≥5 years | Conventional PCR | 983 | 15 |  |  | 15 | 80% | 20% | 0% | 0% |  |  |  |  |  |
| Xue | Prevalence and Genetic Diversity of Human Sapovirus Associated with Sporadic Acute Gastroenteritis in South China From 2013 To 2017 | Journal of Medical Virology | 2019 | China | LMD | Inpatient | <5 years, ≥5 years | Conventional PCR | 569 | 11 |  |  | 11 | 82% | 9% | 9% | 0% |  |  |  |  |  |
| Yandle | Group A Rotavirus Detection and Genotype Distribution Before and After Introduction of a National Immunisation Programme In Ireland: 2015-2019 | Pathogens | 2020 | Ireland | Developed | Mixed | <5 years | RT-qPCR | 11800 | 559 |  |  |  |  |  |  |  |  |  |  |  |  |
| Yoneda | Epidemiological Characteristics of Sapovirus And Human Astrovirus Detected Among Children in Nara Prefecture, Japan, During The 2009/2010-2014/2015 Seasons | Japanese Journal of Infectious Diseases | 2017 | Japan | Developed | Mixed | <5 years, ≥5 years | Conventional PCR | 948 | 71 |  |  | 71 | 79% | 20% | 0% | 1% |  |  |  |  |  |
| Yu | Long-Term Impact of Suboptimal Rotavirus Vaccines on Acute Gastroenteritis in Hospitalized Children in Northern Taiwan | Journal of the Formosan Medical Association | 2018 | Taiwan | LMD | Inpatient | <5 years | Conventional PCR | 837 | 9 |  |  |  |  |  |  |  |  |  |  |  |  |
| Zhang | Emergence of Human Caliciviruses Among Diarrhea Cases in Southwest China | BMC Infectious Diseases | 2016 | China | LMD | Outpatient | <5 years, ≥5 years | Conventional PCR | 1121 | 3 | 319 | 0 | 3 | 67% | 33% | 0% | 0% |  |  |  |  |  |
| Zhu | Analysis of The Aetiology Of Diarrhoea In Outpatients In 2007, Henan Province, China | Epidemiology and Infection | 2013 | China | LMD | Outpatient | <5 years, Mixed | Conventional PCR | 1526 | 29 |  |  |  |  |  |  |  |  |  |  |  |  |
